# Potential antioxidant and cytotoxic impacts of defatted extract rich in flavonoids from* Styphnolobium japonicum* leaves growing in Egypt

**DOI:** 10.1038/s41598-024-68675-2

**Published:** 2024-08-12

**Authors:** Amal M. El‑Feky, Nadia A. Mohammed

**Affiliations:** 1 Pharmacognosy Department, National Research Centre, 33 El Bohouth St. (Former El Tahrir St.), P.O. 12622, Dokki, Giza, Egypt; 2 Department of Medical Biochemistry, National Research Center, 33 El Bohouth St. (Former El Tahrir St.), P.O. 12622, Dokki, Giza, Egypt

**Keywords:** *Styphnolobium japonicum*, Flavonoids, Antioxidant, Anticancer, Biochemistry, Plant sciences, Climate sciences, Biomarkers, Diseases, Medical research

## Abstract

*Styphnolobium japonicum* leaves are considered a rich source of flavonoids, which are the prospective basis for various therapeutic effects. However, there has been a lack of comprehensive cytotoxic studies conducted on these leaves. Therefore, this ongoing investigation aimed to detect and isolate the flavonoids present in *S. japonicum* leaves, and assess their antioxidant and anticancer properties. The defatted extract from *S. japonicum* leaves was analyzed using HPLC, which resulted in the identification of seven phenolics and six flavonoids. Rutin and quercetin were found to be the most abundant. Furthermore, a comprehensive profile of flavonoids was obtained through UPLC/ESI–MS analysis in negative acquisition mode. Fragmentation pathways of the identified flavonoids were elucidated to gain relevant insights into their structural characteristics. Furthermore, genistein 7-*O*-glucoside, quercetin 3-*O*-rutinoside, and kaempferol 3-*O*-*α*-L-rhamnopyranosyl-(1 → 6)-*β*-D-glucopyranosyl-(1 → 2)-*β*-D-glucopyranoside were isolated and characterized. The defatted extract rich in flavonoids exhibited significant antioxidant, iron-reducing, free radicals scavenging impacts, and remarkable cytotoxicity against the liver cell line (IC_50_ 337.9μg/ mL) and lung cell line (IC_50_ 55.0 μg/mL). Furthermore, the antioxidant and anticancer capacities of the three isolated flavonoids have been evaluated, and it has been observed that their effects are concentration-dependent. The findings of this research highlight the promising impact of flavonoids in cancer therapy. It is recommended that future scientific investigations prioritize the exploration of the distinct protective and therapeutic characteristics of *S. japonicum* leaves, which hold significant potential as a valuable natural resource.

## Introduction

*Styphnolobium japonicum*, commonly known as *Sophora japonica* L., is a member of the Fabaceae family and is found extensively in Egypt, China, and Japan^1,2^. In traditional medicine, different parts of *S. japonicum* have been utilized to treat conditions such as bleeding, arteriosclerosis, and hypertension^3–5^. Previous research has primarily focused on analyzing the chemical constituents present in the leaves, fruits, and barks of *S. japonicum* includes sterols, terpenes, phenolic acids, and flavonoids^6–8^. These compounds exhibit various pharmacological properties, including antioxidant activity^9^, anti-inflammatory properties^10^, and cytotoxic impacts^11,12^. According to Nardini and Garaguso^13^, *S. japonicum* has been recognized as a plentiful reservoir of flavonoids, which have the potential to serve as a foundation for diverse therapeutic benefits^14,15^. Numerous flavonoids and polyphenolic compounds, including kaempferol, quercetin, rutin, isorhamnetin, and sophoricoside in *S. japonicum* is thought to play a pivotal role in its antioxidant capacity, as indicated by Zhu et al.^9^, He et al.^12^, and Kite et al.^16^. Furthermore, various flavonol glycosides and abundant isoflavonoid glycosides mainly derived from the isoflavone genistein have been identified in the leaves, as genistein 7-*O*-*ꞵ*-D-glucopyranoside-4′-*O*-(6′′′-*O*-R-L-rhamnopyranosyl)-*ꞵ*-sophoroside and genistein 7-*O*-R-L-rhamnopyranoside-4′-*O*-(6′′′-*O*-R-L-rhamnopyranosyl)-*ꞵ*-sophoroside in addition to quercetin 3-*O*-*ꞵ*-D-glucopyranoside, and kaempferol 3-*O*-*ꞵ*-D-glucopyranoside^17^.

In 2018, cancer claimed the lives of 9.6 million individuals worldwide, making it as the second most significant contributor to global mortality. However, projections indicate that by the year 2030, the global scale will witness a significant rise in cancer-related fatalities, reaching a staggering 11.5 million. Given its lethal nature, extensive international efforts are underway to combat this disease^18^. Numerous studies have provided evidence that plants containing antioxidants possess the potential to significantly reduce the risk of various forms of cancer^19–21^. This suggests that antioxidants have the potential to effectively inhibit cancer metastasis with minimal toxicity and acceptable safety levels^22^.

Several research studies have delved into the potential anticancer effects of flavonoids. These studies, conducted by El-Feky et al.^23^, Kopustinskiene et al.^24^ and Mavundza et al.^25^ have documented various mechanisms through which flavonoids exert their effects. One such mechanism is the safeguarding of DNA from oxidative damage. Flavonoids also possess the capacity to neutralize carcinogens and inhibit the activation of genes that promote procarcinogenic substances. Additionally, flavonoids activate systems that facilitate xenobiotic detoxification, further contributing to their anticancer properties. In vitro studies have further confirmed that flavonoids can alter the action of several enzymes in mammals, including kinases, phospholipases, ATPases, lipooxygenases, cyclooxygenases, and phosphodiesterases. The discoveries made in this research underscore the significant promise of flavonoids as potential means of cancer prevention and treatment.

In light of the abundant presence of phytoconstituents, particularly flavonoids, in the leaves of *S. japonicum*, the primary objective of this investigation was to identify and isolate flavonoidal compounds from the defatted extract of *S. japonicum* leaves. Furthermore, the study sought to assess the antioxidant and anticancer potential of the defatted extract, and the capability of the three obtained flavonoids as free radical scavenger and cytotoxic agents. This comprehensive approach was undertaken to improve our knowledge of phytochemistry and its correlation with the cytotoxicity of *S. japonicum* leaves.

## Materials and methods

### Chemicals

The HPLC requirements and all tested compounds were of the highest analytical purity. The phenolic compounds and flavonoids in this study have been procured from Sigma-Aldrich Co. USA. While DPPH and ABTS have been supplied by Aldrich Chemie, Germany. Dulbecco's Modified Eagle's Medium (DMEM), penicillin/streptomycin and L-glutamine were purchased from Gibco BRL, CA, USA. Fetal bovine serum (FBS), 3-(4, 5-dimethylthiazol-2-yl)-2, 5-diphenylthiazolium bromide (MTT), were provided by Sigma-Aldrich (St. Louis, MO, USA). The cancer cell lines employed in this research, including the liver cell line (hepG2) and lung cell line (A549), were obtained from NAWAH scientific research center.

### Cell culture management

The evaluation of cytotoxicity was conducted within controlled and sterile conditions utilizing a Laminar flow biosafety cabinet Class II A2. The cells were maintained in Dulbecco's Modified Eagle's Medium supplemented with 10% heat-inactivated fetal bovine serum, 100 U/ml penicillin, and 100 μg/ml streptomycin sulfate at room temperature in humid incubators containing 5% CO_2_. Once the cells reached approximately 80% proliferation, they were subcultured using trypsin–EDTA solution. The cells were harvested during the logarithmic phase of growth. The investigated plant extract was dissolved in DMSO and stored at − 20 °C. For each experiment, the solution stocks were combined with the culture medium to achieve the desired final concentrations.

### Plant collection and extraction

Leaves of *S. japonicum* have been collected in October 2022 from Orman Garden, Giza, Egypt. Coordinates 30°01′45″N 31°12′47″E/30.02917°N 31.21306°E/30.02917; 31.21306. The plant collection and use was in accordance with all the relevant guidelines, approved under NO 3445062023 by the Medical Ethical Committee of the National Research Centre in Egypt. The verification of these leaves was conducted by Mrs. Trease Labibm, a Plant Taxonomy Consultant at the Ministry of Agriculture. A specimen has been placed in the herbarium of NRC, Cairo, Egypt (Voucher NO. M215). The gathered leaves were subjected to air drying and subsequent grinding. Following the methodology outlined by Otsuka^26^, the extraction and solvent solvent partition procedure was employed. In summary, 400 g of powdered leaves were subjected to cold maceration using 80% aqueous methanol (2 L X 8). The resulting extract was concentrated using rotatory evaporator (Heidolph, Germany).

at 50 °C to yield dark green total extract weighing 32 g. The concentrated extract was then partitioned successively with n-hexane (200 ml × 5), yielding 6.5 g of defatted fraction. After that, the remaining extract was suspended in methanol and the resulting defatted methanolic extract, weighed 23 g post-evaporation. This particular defatted extract was then used for both phytochemical and biological assessments.

### Phytochemical investigation

#### Quantification of total phenolics and flavonoids

The total phenolic content was estimated in the defatted extract from *S. japonicum* leaves using the Folin–Ciocalteu method, as informed by El-Feky et al*.*^27^. The value obtained was stated as gallic acid equivalent. Additionally, the aluminum chloride method, through Baba et al.^28^, was employed to assess the total flavonoids, with the concentration of flavonoids being demonstrated as rutin equivalent in the fraction. All outcomes were expressed as mean ± S.D.

#### Phenolic acids and flavonoids profile

Identification of various phenolic compounds and flavonoidal compounds in the defatted extract from *S. Japonicum* leaves were conducted through HPLC analysis using Agilent Technologies 1100 series liquid chromatography. The HPLC system was furnished with an autosampler and a Diode-Array Detector (DAD). The mobile phase comprised from solvent system of acetonitrile (solvent A) and 2% acetic acid in water (v/v) (solvent B). The flow rate was kept at 0.8 m min^−1^ for a total run time of 70 min and the gradient program was as follows: 100% B to 85% B in 30 min, 85% B to 50% B in 20 min, 50% B to 0% B in 5 min and 0% B to 100% B in 5 min.^29^. The detection of benzoic acid, cinnamic acid derivatives, and flavonoids was achieved at wavelengths of 280, 320, and 360 nm, respectively. The peaks were identified by examining their retention periods, UV absorbance, and comparison to reference compounds. Furthermore, the UPLC/ESI–MS analysis was conducted in negative ion mode using a XEVO TQD triple quadrupole instrument (Waters Corporation, Milford, MA 01,757 USA). The mass spectrometer utilized an ACQUITY UPLC-BEH column with C18 as the stationary phase (1.7 μm, 2.1 × 50 mm)^30^. The mass spectrum of the eluted compounds was determined within the *m/z* range of 40 to 700 using MassLynx 4.1, SCN 888 software (https://www.waters.com/) and confirming the results through comparing their retention times and mass fragments with those reported previously.

#### Isolation of main flavonoids

The defatted extract of *S. japonicum* leaves (five grams) were chromatographed on a glass column having dimension of 15 × 4 cm, containing 25 g of silica gel. Elution was performed using a gradient of chloroform and methanol. Successive fractions of 100 ml each have been obtained and concentrated to 5 ml, resulting in 20 fractions being produced. The fractions have been subsequently analyzed on thin-layer chromatography (TLC) plates utilizing a developing system composed of chloroform and methanol in a volumetric ratio of 9:1. The fractions that exhibited identical chromatographic profiles were combined. The spots that exhibited a yellow color upon treatment with ammonia and AlCl_3_ spray reagent were isolated and purified several times on preparative TLC using ethyl acetate:ethanol (9.8:0.2) and chloroform:ethyl acetate (4 :1)^31^. The isolated compounds were further characterized through various spectroscopic analyses; UV–Visible Spectrophotometer (UVD–3500, Labomed, Inc.), Mass Spectrometer (Finnigan Model 3200 Mass spectrometer at 70 eV), NMR (JEOL EX-500 spectroscopy, Tokyo, Japan), and by comparing them with previously reported data. The complete hydrolysis of the isolated flavonoid glycoside has been employed following the technique reported by Harborne et al.^32^. In this procedure, the compound was dissolved in 5ml of dilute HCl in 80% methanol and then subjected to heating at 100ºC for duration of two hours. To extract the aglycone, ethyl acetate has been incorporated to the mixture, while the remaining aqueous phase contained the sugar. The sugar component was subsequently identified using Paper Chromatography on Whatman No.1 paper sheets from England, alongside standard sugars. For the development of the chromatogram, a solvent system comprising of n-butanol, acetic acid, and water in a ratio of 4:1:5 has been employed. Descending technique was adopted for this purpose. After spraying the chromatogram by aniline-phthalate and heat it at 110 °C for five minutes, the sugar bands were detected. This methodology was based on the work of Stahl^33^.

### Biological evaluation

#### Antioxidant assessment

The assessment of total antioxidant capacity (TAC) and total iron reducing power was conducted through the procedures outlined by Prieto et al.^34^ and Oyaizu^35^, correspondingly. Furthermore, the ability of the defatted extract of *S. japonicum* leaves (10:100 g) to scavenge DPPH and ABTS radicals was tested at serial concentrations by means of the methods recommended by Rahman et al.^36^ and Arnao et al.^37^.

#### Cytotoxicity evaluation

The cytotoxic evaluation of the defatted extract of *S. japonicum* leaves against hepG2 and A549 was conducted using the MTT assay, as described by Mosmann^38^. Serial dilutions of the defatted extract were used to treat the cells for a duration of forty-eight hours, with final concentrations of (400, 200, 100, 50, 10, 1, 0.1 ug/ml). The potency was compared with reference drug (Doxorubicin). The percentage of cell viability was obtained by calculating for the ratio of the absorbance of the tested plant extract to the absorbance of the control, multiplied by 100. Additionally, IC_50_ of the plant extract was determined using Sigma Plot software version 11 (Spw.exe) (https://sigmaplot.software.informer.com/11.0/).

$$ {\text{Viability }} = {\text{ absorbance of the plant extract}}/{\text{absorbance of control }} \times { 1}00, $$

$$ {\text{Cytotoxicity }} = { 1}00 - {\text{ viability}}. $$

### Statistics

Statistical valuations of the tested parameters were performed using SPSS 9.05 (USA). Values are represented by mean ± SE of three replicates.

### Ethical approval and consent to participate

The research study has been approved by Medical Ethical Committee of National Research Centre in Egypt (NO 3445062023).

## Results

### Quantification of total phenolics and flavonoids

Quantification of total phenolics and flavonoids was conducted on the defatted extract of *S. japonicum* leaves resulting in values of 248.41 ± 0.23 mgGAE/g and 735.21 ± 0.19 mg rutin/g respectively. These findings confirm the significant presence of total phenolics and flavonoids in the leaves of *S. japonicum*, which create the basis for a wide range of biological functions.

### Identification of phenolic acids and flavonoids

**Figure 1:**
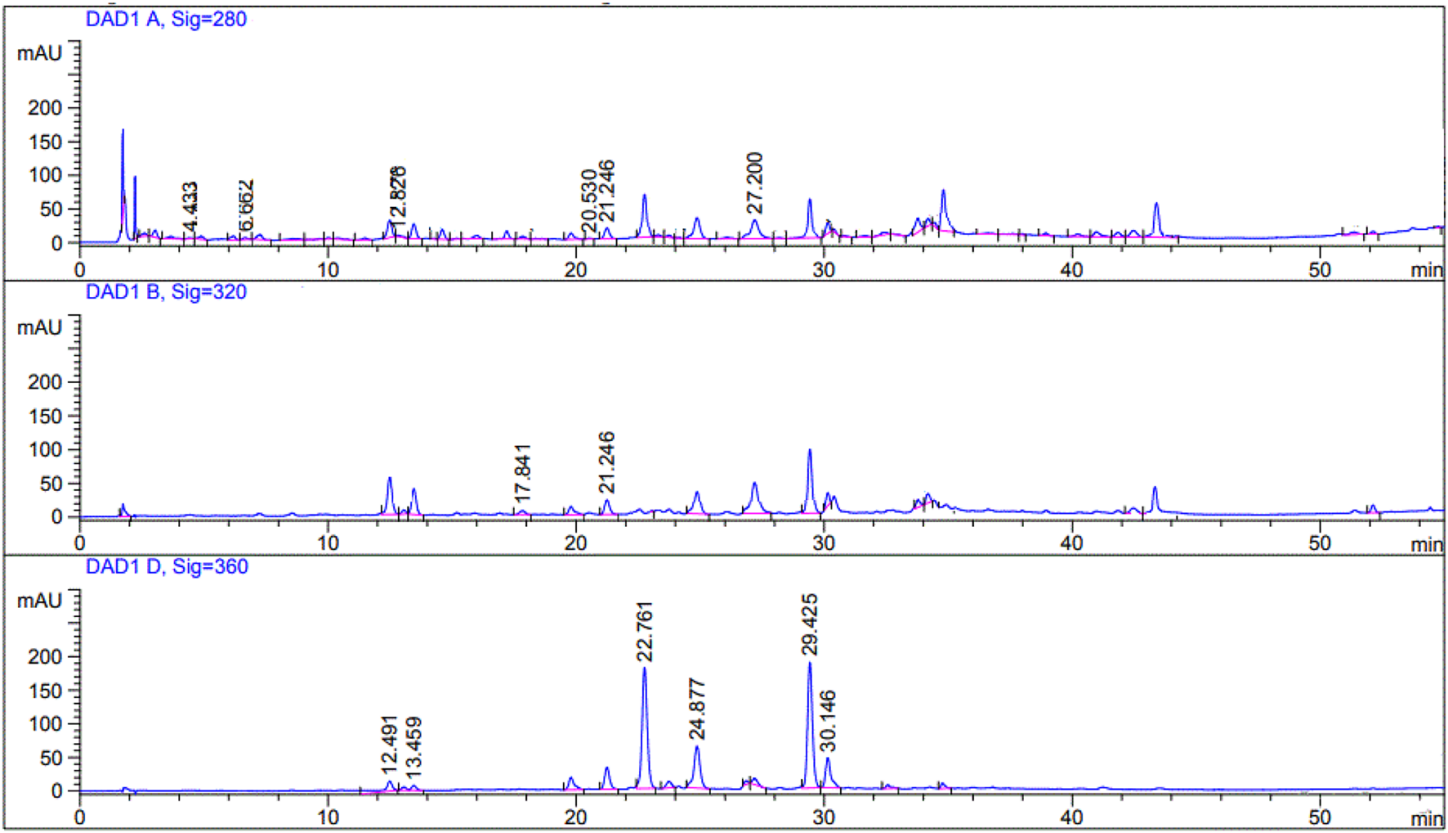
HPLC chromatogram for phenolic acids and flavonoids in the flavonoids-rich fraction of *S. Japonicum* (L.) Schott. Leaves.

**Figure 2:**
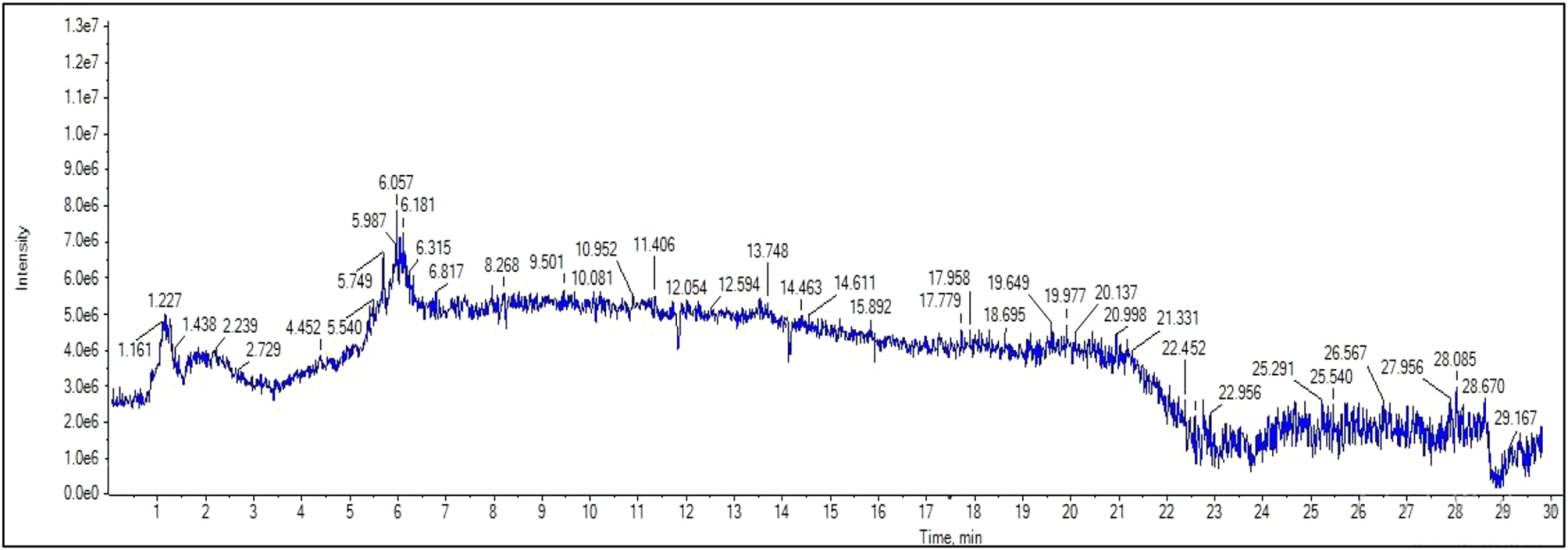
UPLC/ESI–MS chromatogram for the flavonoids profile in the flavonoids-rich fraction of *S. Japonicum* (L.) Schott. leaves in negative ion mode.

HPLC was conducted on the defatted extract of *S. japonicum* leaves, with the objective of determining the different phenolic acids and flavonoids present, as depicted in Table 1 and Fig. 1. The findings of the analysis unveiled the existence of seven phenolic acids and six flavonoids. It is worth mentioning that gentisic acid (R_*t*_ 12.82) exhibited the highest concentration among the phenolic acids, measuring 204.11 ug/g. In terms of flavonoids, rutin (R_*t*_ 22.76) and quercetin (R_*t*_ 29.42) were the primary compounds identified, with concentrations of 637.24 ug/g and 478.34 ug/g, respectively. Furthermore, the negative ion mode UPLC/ESI–MS analysis was performed for characterization of the flavonoids in *S. japonicum* leaves, revealing the comprehensive profile of flavonoids present. The findings are visually represented in Fig. 2, while a succinct summary of the 33 identified flavonoids can be found in the Table 2. Based on molecular weight, mass fragmentation, and previous research, nine free aglycones were identified as luteolin, dihydrokaempferol, kaempferol, kaempferide, 5-deoxykampferol, genistein, quercetin, myricetin, and isorhamnetin. Thirteen flavonoid mono glycosides were also detected, including luteolin *O*-glucoside, 2'-hydroxygenistein 7-*O*-glucoside, apigenin 7-*O*-glucoside^*^, myricetin 3-*O*-galactoside, isorhamnetin 3-*O*-glucoside, quercetin 3-*O*-rhamnoside, Isoquercitrin, rutin, genistein 7-*O*-glucoside, kaempferol 3-*O*-sophoroside, kaempferol 7-*O*-rhamnoside, Kaempferol 3-*O*-rutinoside, and Kaempferol 3-*O*-glucoside were characterized. Additionally, seven flavonoid diglycosides were identified as genistein 4'-glucoside-rhamnoside, kaempferol 3,7-di-*O*-rhamnoside, luteolin *O*-glucoside-*O*-rhamnoside, quercetin 3,7-di-*O*-rhamnoside, quercetin 3–3′-diglucoside, isorhamnetin 3,7-diglucoside, and kaempferol 3-*O*-glucoside-3''-rhamnoside. Furthermore, in addition to the aforementioned, two types of dihydroxyflavones were identified, specifically 3′,4′-dihydroxyflavone and 3,7-dihydroxy-3',4'-dimethoxyflavone at Rt 11.95 and 16.47, respectively. As well as an *O*-methylated isoflavone known as biochanin A (R_*t*_ 13.03) and its glucoside, sissotrin (biochanin A 7-*O*-*ꞵ*-D-glucoside, Rt 15.35). It is worth noting that compounds 2, 4, 5, 7, 11–13, 16–21, 24, 26, 27, 29, 30, and 32 were not previously documented to be present in the leaves of *S. japonicum* This discovery adds an interesting dimension to our understanding of the chemical composition of these leaves.

**Table 1:** HPLC identification of phenolic acids and flavonoids in the defatted extract of *S. japonicum* leaves.

λmax/nm	R_*t*_	Compound	Concentration (ug/g)
280	4.43	Gallic acid	99.82
	6.66	Protocatechuic acid	115.76
	12.82	Gentisic acid	204.11
	20.53	Syringic acid	ND
	21.24	Vanillic acid	174.87
	27.20	*p*-coumaric acid	98.14
	35.71	Cinnamic acid	ND
320	17.84	Caffeic acid	166.54
	21.24	Ferulic acid	194.78
	23.79	Sinapic acid	ND
360	12.49	Catechin	249.13
	13.45	Apigenin	324.21
	18.24	Naringenin	ND
	22.76	Rutin	637.24
	22.89	hesperidin	ND
	23.16	Naringin	ND
	24.87	Apigenin 7-*O*-glucoside	422.37
	29.42	Quercetin	478.34
	30.14	Kaempferol	357.46

*ND* Not detected, *R*_*t*_ retention time.

**Table 2:** UPLC/ESI–MS analysis (negative mode) of flavonoids in the defatted extract of *S. japonicum* leaves.

No	R_*t*_ (min)	Tentative identification	M.F	M.W	Observed mass (Da)	Calculated mass (Da)	Error (ppm)	Product ions (*m/z*)	Reference
1	6.39	Luteolin*^r^	C_15_H_10_O_6_	286	285. 2402	285. 2390	1.2	267[M-H-H_2_O], 257[M-H–CO], 241[M-H-CO_2_], 217 [M-H-C_3_O_2_], 175[M-H-C_3_O_2_ -C_2_H_2_O], 151[1,3A^−^], 133[1,3B^−^], 148[0,2A^−^], 136 [0,2B^−^]	^39^
2	8.50	Dihydrokaempferol	C_15_H_12_O_6_	288	287.2530	287. 2521	0.9	270 [M-H-OH], 259[M-H–CO], 257[M-H-CH_2_O], 245[M-H-C_2_H_2_O], 241 [M-H–CO-H_2_O]	
3	9.21	Kaempferol*^f^	C_15_H_10_O_6_	286	285.2309	285.2312	-0.3	268 [M-H-OH], 257[M-H–CO], 255[M-H-CH_2_O], 243[M-H-C_2_H_2_O], 239 [M-H–CO-H_2_O], 151[1,3A^−^], 133[1,3B^−^], 164[0,2A^−^], 120 [0,2B^−^]	^17^
4	9.55	Kaempferide	C_16_H_12_O_6_	300	299.2629	299.2613	1.6	284[M-H-CH_3_], 267 [M-H-CH_3_-OH],151[1,3A^−^], 147[1,3B ^−^]	
5	10.94	5-Deoxykampferol	C_15_H_10_O_5_	270	269.2376	269.2371	0.5	252 [M-H-OH], 241[M-H–CO], 239[M-H-CH_2_O], 227[M-H-C_2_H_2_O], 223 [M-H–CO-H_2_O]	
6	11.19	Genistein*^f^	C_15_H_10_O_5_	270	269.2426	269.2413	1.3	241[M-H–CO], 255 [M-H-CO_2_], 197 [M-H–CO-CO_2_],171 [M-H–CO-CO_2_-C2H_2_]	^17^
7	11.95	3,7-Dihydroxy 3',4'-dimethoxyflavone	C_17_H_14_O_6_	314	313.2932	313.2921	1.1	298 [M-H-CH_3_], 283[M-H-CH_3_-CH_3_], 255[M-H-CH_2_O]	
8	12.47	Genistein 4'-glucoside-rhamnoside *^f,s^	C_27_H_30_O_14_	578	577.1123	577.1107	1.6	431[M-H-Rham], 269 [M-H-Rham-Glu], 241[M-H-Rham-Glu -CO], 255 [M-H-Rham-Glu-CO_2_], 197 [M-H-Rham-Glu-CO-CO_2_], 171 [M-H-Rham-Glu-CO-CO_2_ -C2H_2_]	^17^
9	13.03	Sissotrin (biochanin A 7-*O*-*ꞵ*-D-glucoside) *^s^	C_22_H_22_O_10_	446	445 0.0246	445. 0239	0.7	283[biochanin A], 268 [biochanin A-H-CH_3_], 267[biochanin A-H-CH_3_-H], 240 [biochanin A-H-CH_3_-CO], 224 [biochanin A-H-CH_3_-CO_2_], 239 [biochanin A-H-CH_3_-CO–H], 223 [biochanin A-H-CH_3_-CO_2_-H]	^3^
10	13.76	Quercetin*^f^	C_15_H_10_O_7_	302	301.2404	301.2411	-0.6	273[M-H–CO], 255[M-H–CO-H_2_O], 179, 151[1,3A^−^]	^17^
11	13.97	Kaempferol dipentoside	C_27_H_30_O_14_	578	577.0287	577.0290	-0.3	431[M-H-pentose], 285[M-H-2 pentose], 255[M-H-2 pentose-CH_2_O]	
12	14.16	Luteolin hexoside	C_21_H_20_O_11_	448	447 0.0282	447 0.0290	-0.8	401[M-H-H_2_O-CO], 285[M-H-hexose], 267[M-H-hexose-H_2_O],	
13	14.45	2'-Hydroxygenistein hexoside	C_21_H_20_O_11_	448	447.1014	447.1005	0.9	285[M-H-hexose], 269 [Genistein] 241[Genist-H–CO], 255 [Genist-H-CO_2_], 197 [Genist–H–CO-CO_2_],171 [Genist-H–CO-CO_2_-C2H_2_]	
14	15.05	Apigenin 7-*O*-glucoside*^r^	C_21_H_20_O_10_	432	431.1067	431.1056	1.1	269 [M-H-Glu], 225 [Apig-H-CO2], 227 [Apig-H-C_2_H_2_O], 183[Apig-H-C_2_H_2_O-CO_2_], 151 [1,3A^−^], 117 [1,3B^−^]	^5^
15	15.35	Biochanin A*^f,r^	C_16_H_12_O_5_	284	283.0691	283.0684	0.7	268 [M-H-CH_3_], 267[M-H-CH_3_-H], 240 [M-H-CH_3_-CO], 224 [M-H-CH_3_-CO2], 239 [M-H-CH_3_-CO–H], 223 [M-H-CH_3_-CO_2_-H]	^40^
16	15.82	Myricetin	C_15_H_10_O_8_	318	317.2416	317.2431	-1.5	299[M-H-H_2_O], 289[M-H–CO], 259[M-H-2CHO]	
17	16.47	3′,4′-Dihydroxyflavone	C_15_H_10_O_4_	254	253.0584	253.0579	0.5	235[M-H-H_2_O], 225[M-H–CO], 209[M-H-CO_2_], 185 [M-H-C_3_O_2_]	
18	16.96	Isorhamnetin	C_16_H_12_O_7_	316	315.0601	315.0589	1.2	300[M-H-CH_3_], 151[1,3A^−^], 107	
19	17.31	Myricetin hexoside	C_21_H_20_O_13_	480	479.0915	479.0904	1.1	317 [M-H- hexose],299[Myr-H-H_2_O], 289[Myr-H–CO], 259[Myr-H-2CHO]	
20	17.98	Isorhamnetin hexoside	C_22_H_22_O_12_	478	477.0413	477.0406	0.7	315[M-H-hexose], 300[M-H-hexose-CH_3_]	
21	18.92	Quercetin pentoside	C_21_H_20_O_11_	448	447.2421	447.2410	1.1	301[M-H-pentose], 271[M-H-pentose-CH_2_O], 255[M-H-pentose-CO-H_2_O]	
22	20.71	Isoquercitrin (quercetin 3-*O*-glucoside) *^f^	C_21_H_20_O_12_	464	463.4356	463.4361	-0.5	301[M-H-Glu], 271[M-H-Glu-CH_2_O], 255[M-H-Glu-CO-H_2_O]	^17^
23	22.64	Rutin (quercetin 3-*O*-rutinoside) *^f , L^	C_27_H_30_O_16_	610	609.5179	609.5175	0.4	301[M-H-Rut], 271[M-H-Rut-CH_2_O], 255[M-H-Rut-CO-H_2_O], 179, 151[1,3A^−^]	^1,17^
24	23.28	Luteolin hexoside pentoside	C_27_H_30_O_15_	594	593.1064	593.1056	0.8	447 [M-H-pentose],431[M-H-hexose], 285[M-H- pentose-hexose]	
25	23.81	Genistein 7-*O*-glucoside *^f^	C_21_H_20_O_10_	432	431.1057	431.1064	-0.7	269 [M-H-Glu], 241[Gen-H–CO], 255 [Gen-H-CO_2_], 197 [Gen-H–CO-CO_2_], 171 [Gen-H–CO-CO_2_-C2H_2_]	^17^
26	24.31	Kaempferol 3-*O*-sophoroside	C_27_H_30_O_16_	610	609.7142	609.7132	1.0	449, 429, 285[M-H-Soph], 268 [M-H-Soph-OH], 257[M-H-Soph-CO], 255[M-H-Soph-CH_2_O], 243[M-H-Soph-C_2_H_2_O], 239 [M-H-Soph-CO-H_2_O]	
27	24.44	Quercetin dipentoside	C_27_H_30_O_15_	594	593.5225	593.5211	1.4	447[M-H-pentose], 301[M-H-2 pentose], 273[M-H-2 pentose-CO], 255[M-H-2pentose-CO-H_2_O]	
28	24.51	Kaempferol 7-*O*-rhamnoside*^s^	C_21_H_20_O_10_	432	431.3793	431.3782	1.1	285[M-H-Rham], 268 [M-H-Rham-OH], 257[M-H-Rham-CO], 255[M-H-Rham-CH_2_O],243[M-H-Rham-C_2_H_2_O], 239 [M-H-Rham-CO-H_2_O]	^17^
29	25.97	Quercetin dihexoside	C_27_H_30_O_17_	626	625.5208	625.5213	-0.5	463[M-H-hexose], 301[M-H-2 hexose], 273[M-H-2 hexose -CO], 255[M-H-2 hexose -CO-H_2_O]	
30	26.44	Isorhamnetin dihexoside	C_28_H_32_O_17_	640	639. 0446	639. 0440	0.6	477[M-H-hexose], 315[M-H-2 hexose], 300[M-H-2 hexose-CH_3_]	
31	27.04	Kaempferol 3-*O*-glucoside-3''-rhamnoside *^f^	C_27_H_30_O_15_	594	593 0.0193	593 0.0184	0.9	447[M-H-Rham], 285[M-H-Rham-Glu], 268 [M-H-Rham-Glu-OH], 257[M-H-Rham-Glu-CO], 255[M-H-Rham-Glu-CH_2_O],243[M-H-Rham-Glu-C_2_H_2_O], 239 [M-H-Rham-Glu-CO-H_2_O]	^16^
32	27.86	Kaempferol rutinoside	C_27_H_30_O_15_	594	593.5236	593.5231	0.5	285[M-H-Rut], 255[M-H-Rut-CH_2_O], 227[M-H-Rut-2CHO]	
33	28.11	Kaempferol 3-*O*-glucoside*^f^	C_21_H_20_O_11_	448	447.3879	447.3867	1.2	285[M-H-Glu], 268 [M-H-Glu-OH], 257[M-H- Glu-CO], 255[M-H-Glu-CH_2_O],243[M-H-Glu-C_2_H_2_O], 239 [M-H-Glu-CO-H_2_O]	^17^

*Compounds have been previously documented in (f) fruits, (s) seeds, and (r) roots of *S. japonicum.*

### Structural elucidation of the isolated flavonoids

Compound 1 was acquired as 29mg pale-yellow powders after isolating it from a fraction of chloroform–methanol (80:20, *v/v*). Its melting point was determined to be 165 °C. UV of compound 1 inmethanol exhibited absorption maxima at wavelengths of 245 nm, 272 nm, and 356 nm, indicating its flavonoid structure. When Compound 1 was treated with methanol and sodium methoxide (MeOH + NaOMe), a bathochromic shift in band I was detected at wavelengths of 260 nm, 273 nm, and 370 nm, suggesting the existence of free hydroxy group at C-4'. Similarly, treatment with methanol and aluminum chloride (MeOH + AlCl_3_) caused bathochromic shift at wavelengths of 280 nm, 359 nm, and 394 nm, signifying the occurrence of free hydroxyl group at either position C-3 or C-5. Further treatment with methanol, aluminum chloride, and hydrochloric acid (MeOH + AlCl_3_/HCl) showed a bathochromic shift at wavelengths of 279 nm, 358 nm, and 393 nm, while treatment with methanol and sodium acetate (MeOH + NaOAc) did not cause any change in absorbance in band II, suggesting substitution of the 7-hydroxyl group. However, a bathochromic shift was observed in band I, confirming the presence of a free hydroxyl group at position 4'. Finally, treatment with methanol, sodium acetate, and boric acid (MeOH + NaOAc/H_3_BO_3_) did not show any change in UV absorbance, indicating the absence of *ortho*-dihydroxyl groups. The infrared (IR) data obtained from the KBr/cm^-1^ spectrum exhibited prominent absorption peaks at specific wavenumbers. These included a strong absorption band at 3225 cm^−1^, that corresponds to OH groups. Another significant absorption band was observed at 2927 cm^−1^, indicating the stretching of both methylene (CH_2_) and carbon-hydrogen (C-H) bonds. Additionally, a distinct absorption peak at 1718 cm^−1^ designated to conjugated carbonyl groups. The spectrum also displayed an absorption band at 1657 cm^-1^, which could be ascribed to the aromatic carbon–carbon (C=C) bonds. Furthermore, absorption peaks at 1437 cm^−1^ and 1134 cm^−1^ were observed, indicating the stretching of carbon–oxygen-hydrogen (C–O–H) and carbon–oxygen-carbon (C–O–C) bonds, respectively. ESI–MS analysis provided molecular ion with *m/z* 432. This value corresponds to the estimated molecular formula of C_21_H_20_O_10_. Notably, a distinctive product ion was detected at *m/z* 270, that referred to genistein aglycone. Additionally, a product ion at *m/z* 134 was observed, correspondent to a basic fragmentation product resulting through the C-ring cleavage, specifically involving the presence of HO–C≡C–(C_6_H_4_)–OH. The ^1^H-NMR analysis (CD_3_OD, 500 MHz) displayed signals at 7.99 ppm (1H, *s*, H-2), 6.87 ppm (1H, *d*, *J* = 2.5 Hz, H-6), 6.54 ppm (1H, *d*, *J* = 2.5 Hz, H-8), 7.42 ppm (2H, *dd*, *J* = 7.8 Hz, H-2', H-6'), 6.89 ppm (2H, *dd*, *J* = 7.8 Hz, H-3', H-5'). The anomeric proton was observed at 4.89 ppm (1H, *d*, H-1''), along with glycosidic protons at 3.54–3.26 ppm. The ^13^C-NMR analysis (CD_3_OD, 125 MHz) showed signals at 154.3 ppm (C-2), 125.6 ppm (C-3), 179.8 ppm (C-4), 162.3 ppm (C-5), 98.6 ppm (C-6), 162.8 ppm (C-7), 96.4 ppm (C-8), 158.8 ppm (C-9), 104.8 ppm (C-10), 132.9 ppm (C-1'), 131.4 ppm (C-2', C-6'), 116.2 ppm (C-3', C-5'), 160.1 ppm (C-4'), 100.8 ppm (C-1''), 75.6 ppm (C-2''), 74.8 ppm (C-3''), 69.5 ppm (C-4''), and 77.4 ppm (C-5''). The findings were in line with the outcomes documented by Ichige et al.^41^. The glycosidic hydrolysis of the isolated compound confirmed the presence of glucose within its structure. Through a comprehensive review of previous studies on flavonoid chemistry^42,43^, and an analysis of spectroscopic results from the literature, the isolated compound was identified as genistein 7-*O*-*ꞵ*-glucoside (genistin). This identification was further supported by UPLC/ESI–MS analysis. The compound was previously discovered in the pericarps of *S. japonicum* by Tang et al.^44^.

Compound 2 was obtained from a fraction of chloroform–methanol (70:30, *v/v*) as 34mg yellow needle-shaped crystals with a melting point of 242 °C. The UV spectrum of the isolated compound in methanol revealed absorption peaks at 254 nm, 263 nm (shoulder), and 351 nm, indicating the presence of a flavonol nucleus. When the isolated compound was treated with methanol and sodium methoxide, a bathochromic shift was observed at 267 nm, 299 nm, and 389 nm, confirming the existence of polyhydroxyl groups. Further treatment with methanol and aluminum chloride resulted in a bathochromic shift at 274 nm, 305 nm (shoulder), 335 nm, and 436 nm, that was assigned to the free hydroxyl group at either C-3 or C-5. Treatment with methanol, aluminum chloride, and hydrochloric acid showed a bathochromic shift at 273 nm, 304 nm (shoulder), 336 nm, and 433 nm. Additionally, treatment with methanol and sodium acetate resulted in a bathochromic shift at 275 nm, 326 nm (shoulder), and 375 nm, that was recognized to free 4'-hydroxyl group. Finally, treatment with methanol, sodium acetate, and boric acid showed a bathochromic shift at 261 nm, 305 nm (shoulder), and 385 nm, indicating the presence of a 3',4'-dihydroxy group. IR analysis of the isolated compound, recorded using a potassium bromide (KBr) pellet, exhibited strong absorption bands at 3416 cm^−1^ (OH stretching), 2896 cm^−1^ (CH2 and C-H stretching), 1724 cm^−1^ (C=O conjugation), 1643 cm^−1^ (aromatic C=C stretching), 1512 cm^-1^ (C–O–H stretching), and 1078 cm^−1^ (C–O–C stretching). ESI–MS demonstrated the presence of a molecular ion at *m/z* 610, that corresponds to the calculated molecular formula of C_27_H_30_O_16_, The characteristic product ion, identified as *m/z* 302 (C_15_H_10_O_7_), was observed after the loss of rutinose from quercetin aglycone. Additionally, a peak at *m/z* 284, consistent to C_15_H_8_O_6_ was detected as a result of water loss. Consequently, ions at *m/z* 274 (C_14_H_10_O_6_) and 246 (C_13_H_10_O_5_) were observed due to sequential CO loss from quercetin (*m/z* 302). Furthermore, the presence of an ion peak at *m/z* 122 (C_7_H_6_O_2_), containing the B ring (cinnamyl system), and *m/z* 180 (C_8_H_4_O_5_), containing the A ring (benzoyl system), was attributed to the cleavage of quercetin aglycone. The ^1^H-NMR data was obtained using a 500 MHz instrument in CD_3_OD solvent. The chemical shifts (δ) in parts per million (ppm) were recorded as follows: 6.44 (1H, *d*, *J* = 2.2 Hz, H-6), 6.37(1H, *d*, *J* = 2.2Hz, H-8), 7.64 (1H, *d*, *J* = 2.8 Hz, H-2'), 6.40 (1H, *d*, *J* = 8.4 Hz, H-5'), 7.62 (1H, *dd*, *J* = 2.8, 8.4Hz, H-6'). The anomeric proton, H-1'', appeared at a chemical shift of 4.96 (1H, doublet). The rutinoside protons were observed in the range of δ 3.12–3.78, and a methyl group (CH_3_-Rhamnose) was detected at δ 1.02 (3H, doublet, *J* = 5.7 Hz). The ^13^C-NMR data was acquired using a 125 MHz instrument in CD_3_OD solvent. The chemical shifts (δ) in parts per million (ppm) were recorded as follows:157.9 (C-2), 136.2 (C-3), 180.2 (C-4),162.8 (C-5), 101.2 (C-6), 165.7 (C-7), 96.1(C-8), 159.3 (C-9), 105.3 (C-10),123.5 (C-1'), 118.4 (C-2'), 146.2 (C-3'), 151.2(C-4'), 116.3 (C-5'), 124.1 (C-6'), 103.2 (C-1''),76.1 (C-2''), 77.8 (C-3''), 71.4 (C-4''), 78.2 (C-5''), 69.5 (C-6''), 101.6 (C-1'''),73.4 (C-2'''), 72.1 (C-3'''), 74.3 (C-4'''), 70.1 (C-5'''), 54.9 (C-6'''). The spectroscopical data were consistent with the findings reported by Yingyuen et al.^45^. The glycosidic hydrolysis of the compound that was isolated resulted in the formation of glucose and rhamnose in the aqueous phase. Through spectral analyses, melting point determination, and comparison with published data, the isolated compound has been established to be rutin (quercetin 3-*O*-rutinoside). This identification was further confirmed by UPLC/ESI–MS analysis. It is worth noting that rutin had previously been isolated from the leaves of *S. japonicum* by Abdelhady et al.^1^. Numerous research studies have demonstrated that *S. japonicum* showcases a relatively elevated concentration of rutin when compared to other bioactive compounds sourced from nature^9^.

Compound 3 was isolated from a fraction of chloroform–methanol (30:70, *v/v*) and obtained as 27mg yellow crystals with a melting point of 185°C. UV of the isolated compound in methanol exhibited absorption maxima at wavelengths of 262, 301, and 352 nm, which are indicative of a flavonol structure. When the isolated compound was treated with MeOH + NaOMe, a bathochromic shift in band I was observed, suggesting the presence of a free OH group at C-4'. Similarly, treatment with MeOH + AlCl_3_ resulted in a bathochromic shift at wavelengths of 274, 309, 351, and 401 nm, indicating the presence of a free OH group at either C-3 or C-5. Further treatment with MeOH + AlCl_3_/HCl yielded absorption peaks at 276, 307, 350, and 399 nm. Additionally, when the isolated compound was treated with MeOH + NaOAc, a bathochromic shift in band II was observed at wavelengths of 276, 320, and 395 nm, confirming the existence of a free OH group at C-7. Finally, treatment with MeOH + NaOAc/H_3_BO_3_ resulted in absorption peaks at 263 and 351 nm. The IR spectrum of the isolated compound recorded using KBr, exhibited strong absorption bands at 3410 cm^-1^ (OH stretching), 2913 cm^−1^ (CH2 and C-H stretching), 1670 cm^−1^ (C=O conjugation), 1605 cm^−1^ and 1528 cm^−1^ (aromatic C=C stretching), 1442 cm^−1^ (C–O–H stretching), and 1120 cm^−1^ (C–O–C stretching). ESI–MS analysis revealed the presence of a molecular ion at *m/z* 756, which corresponds to the molecular formula C_33_H_40_O_20_. Additionally, characteristic fragments were observed at *m/z* 610, indicating the loss of rhamnose, at *m/z* 448, indicating the loss of rhamnose and glucose, and at *m/z* 286, confirming the appearance of kaempherol aglycone after the loss of one rhamnose and two glucose molecules. The structure of the compound has been further clarified through the analysis of its ^1^H-NMR and ^13^C-NMR data. The ^1^H-NMR analysis revealed specific signals at 6.22 ppm (1H, *d*, *J* = 2.1Hz, H-6) and 6.45 ppm (1H, *d*, *J* = 2.1Hz, H-8), which are characteristic of the kaempferol moiety. Additionally, signals at 8.03 ppm (2H, *d*, *J* = 7.9Hz, H-2', H-6') and 7.11 ppm (2H, *d*, *J* = 7.9Hz, H-3', H-5') were also observed, further confirming the presence of the kaempferol moiety. Furthermore, the ^1^H-NMR exhibited three anomeric signals at 5.59 ppm, 5.07 ppm, and 4.89 ppm, indicating the presence of three sugars. Additionally, 15 other signals were observed in the range of 3.56–1.94 ppm, which can be attributed to the glycosidic protons. Moreover, three signals were detected at 12.01 ppm, 11.54 ppm, and 10.97 ppm, suggesting the presence of three free hydroxyl (OH) groups at positions 5, 7, and 4' of the kaempferol molecule. The ^13^C-NMR provided further information about the structure of the compound. Notable carbon signals include 158.74 ppm (C-2), 130.51 ppm (C-3), 165.04 ppm (C-4), 161.74 ppm (C-5), 99.13 ppm (C-6), 163.74 ppm (C-7), 95.76 ppm (C-8), 156.82 ppm (C-9), 103.41 ppm (C-10), 121.43 ppm (C-1'), 132.11 ppm (C-2'&6'), 117.52 ppm (C-3'&5'), and 159.16 ppm (C-4'). Additionally, signals corresponding to the sugar; 73.58 (C-2‶), 76.34 (C-3‶), 69.12 (C-4‶), 77.54 (C-5‶), 60.34 (C-6‶), 74.62 (C-2‷), 76.81 (C-3‷), 69.55 (C-4‷), 76.12 (C-5‷), 62.77 (C-6‷), 71.01 (C-2‷‵), 71.69 (C-3‷‵), 69.78 (C-4‷‵), 78.01 (C-5‷‵), 59.76 (C-6‷‵). The data were consistent with those previously reported for the kaempferol-3-*O*-glycosidic structure, where kaempherol is linked to three sugars at the C-3 position^43^. The confirmation of glycoside hydrolysis indicated the presence of rhamnose and glucose. Through a comprehensive review of existing literature on the chemical structure of flavonoids^42,43^, and by comparing the physical and spectroscopic results with the available information, the isolated compound was acknowledged as Kaempferol 3-*O*-*α*-L-rhamnopyranosyl-(1 → 6)-*β*-D-glucopyranosyl -(1 → 2)-*β*-D-gluco-pyranoside. This compound was formerly been recognized in the fruits by Tang et al.^46^, but this investigation marks the first time it has been discovered in leaves.

### Antioxidant and cytotoxicity evaluation of the defatted extract

The defatted extract of *S. japonicum* leaves demonstrated impressive total antioxidant capacity and iron reducing power. The values obtained for these properties were 163.79 ± 0.84 mg gallic acid/g and 238.67 ± 0.52μg/ mL, respectively, as presented in Table 3. Additionally, the scavenging activities against DPPH and ABTS radicals were assessed at different concentrations (10-100μg/ml). Notably, the defatted extract of *S. japonicum* leaves exhibited significant scavenging activity, surpassing ascorbic acid, which was used as the standard.

**Table 3:** Antioxidant activity of the defatted extract of *S. japonicum* leaves.

Tested groups	Antioxidant capacity (mg gallic acid/g)	Iron reducing power (μg/mL)	Inhibition percentages (%) at concentration of (μg/ml)					
			DPPH			ABTS		
			10	50	100	10	50	100
Defatted extract	163.79 ± 0.84	238.67 ± 0.52	42 ± 0.08	67 ± 0.14	91 ± 0.09	38 ± 0.21	57 ± 0.05	88 ± 0.10
Ascorbic acid			58 ± 0.11	81 ± 0.04	96 ± 0.06	47 ± 0.12	79 ± 0.13	93 ± 0.06

Values are represented by mean ± SE of three replicates.

In addition, the tested extract exhibited a decline in liver cell viability by 42.3% at 400 ug/ml (resulting in a cytotoxicity of 57.7%) and a significant decrease of 99.6% at 6.25 ug/ml (resulting in a cytotoxicity of 0.5%). Conversely, the viability of lung cells was found to be 28.4% at 100 ug/ml (resulting in a cytotoxicity of 71.6%) and 77.6% at 6.25 ug/ml (resulting in a cytotoxicity of 22.4%), as indicated in Table 4. The IC_50_ values for HepG2 and A549 were calculated as 337.9 and 55.0, respectively.

**Table 4:** Percentage of cell viability and cytotoxicity of the defatted extract of *S. japonicum* leaves against liver and lung cells compared to doxorubicin as standard.

Tested group	Tested cell line	liver cells (HepG2)								lung cells (A549)					
	Concentration (ug/ml)	400	200	100	50	25	12.5	6.25	IC_50_%	100	50	25	12.5	6.25	IC_50_%
Defatted extract	% Cell viability	42.3	68.6	81.3	91.2	94.9	99.3	99.6	337.9	28.4	52.4	63.9	71.8	77.6	55.0
	% Cytotoxicity	57.7	31.4	18.7	8.8	5.1	0.7	0.5		71.6	47.6	36.1	28.2	22.4	
Doxorubicin	% Cell viability	10.3	12.8	16.1	30.4	37.5	56.7	64.3	27.65	10.3	24.4	39.0	59.7	69.2	27.02
	% Cytotoxicity	93.5	89.2	83.6	69.2	58.6	43.8	35.6		89.7	75.6	58.4	36.5	29.4	

### Antioxidant and cytotoxicity evaluation of the isolated flavonoids

The antioxidant impact of the three isolated flavonoids has been evaluated by means of DPPH and ABTS scavenging assays. Table 5 demonstrated that these flavonoids possess great through scavenging the free radicals in concentration-dependent manner, ranging from 10 to 100 μg/ml. Out the three flavonoids, rutin unveiled the greatest DPPH and ABTS scavenging activity at 95% and 91%, respectively, followed by genistein 7-*O*-glucoside at 93% and 86%, and kaempferol 3-*O*-*α*-L-rhamnopyranosyl-(1 → 6)-*β*-D-glucopyranosyl-(1 → 2)-*β*-D-glucopyranoside at 89% and 85%, respectively, all at 100 μg/ml. Furthermore, the cell viability and cytotoxicity percentages of the isolated flavonoids from *S. japonicum* leaves on liver and lung cells in comparison to the standard doxorubicin were illustrated briefly in Table 6. Where, in liver cells, genistein 7-*O*-glucoside, rutin, and kaempferol 3-*O*-*α*-L-rhamnopyranosyl-(1 → 6)- *β*-D-glucopyranosyl-(1 → 2)-*β*-D-glucopyranoside displayed cytotoxicity levels of 90.49%, 91.36%, and 82.25% at 100 ug/ml, respectively, resulting in IC50 values of 36.87%, 27.03%, and 36.89%, as compared to doxorubicin (IC50 27.65%). Conversely, in lung cells, the cytotoxicity of genistein 7-*O*-glucoside, rutin, and kaempferol 3-*O*-*α*-L-rhamnopyranosyl-(1 → 6)- *β*-D-glucopyranosyl-(1 → 2)-*β*-D-glucopyranoside was 86.03%, 83.45%, and 69.96% at 100 ug/ml, respectively, resulting in IC50 values of 30.63%, 25.51%, and 43.01%, in comparison to doxorubicin (IC50 27.02%).

**Table 5:** Radicals scavenging activity of isolated flavonoids from *S. japonicum* leaves.

Tested compound	Inhibition percentages at concentration of (μg/ml)					
	DPPH			ABTS		
	10	50	100	10	50	100
Genistein 7-*O*-glucoside	43 ± 0.18	79 ± 0.08	93 ± 0.11	41 ± 0.21	65 ± 0.05	86 ± 0.10
Rutin	56 ± 0.09	82 ± 0.10	95 ± 0.03	46 ± 0.21	69 ± 0.05	91 ± 0.10
Kaempferol 3-*O*-*α*-L-rhamnopyranosyl-(1 → 6)- *β*-D-glucopyranosyl-(1 → 2)-*β*-D-glucopyranoside	49 ± 0.12	59 ± 0.18	89 ± 0.06	33 ± 0.21	54 ± 0.05	85 ± 0.10
Ascorbic acid	58 ± 0.11	81 ± 0.04	96 ± 0.06	47 ± 0.12	79 ± 0.13	93 ± 0.06

Values are represented by mean ± SE of three replicate.

**Table 6:** Percentage of cell viability and cytotoxicity of the isolated flavonoids of *S. japonicum* leaves against liver and lung cells compared to doxorubicin as standard.

Tested group	Tested cell line	liver cells (HepG2)						lung cells (A549)					
	Concentration (ug/ml)	100	50	25	12.5	6.25	IC_50_%	100	50	25	12.5	6.25	IC_50_%
Genistein 7-*O*-glucoside	% Cell viability	9.51	32.01	65.98	79.12	88.20	36.87	13.89	31.12	55.04	65.41	75.48	30.63
	% Cytotoxicity	90.49	67.99	34.01	21.04	11.97		86.03	68.97	45.26	34.77	24.52	
Rutin	% Cell viability	8.61	26.13	38.11	54.96	69.88	27.03	16.59	19.71	30.50	42.63	58.04	25.51
	% Cytotoxicity	91.36	73.89	61.54	47.01	29.35		83.45	79.97	69.48	57.38	41.99	
Kaempferol 3-*O*-*α*-L-rhamnopyranosyl-(1 → 6)- *β*-D-glucopyranosyl-(1 → 2)-*β*-D-glucopyranoside	% Cell viability	17.59	44.02	47.18	73.42	89.14	36.89	29.87	55.01	66.12	75.64	87.76	43.01
	% Cytotoxicity	82.25	56.11	53.01	26.78	9.48		69.96	45.14	33.99	24.38	12.29	
Doxorubicin	% Cell viability	16.1	30.4	37.5	56.7	64.3	27.65	10.3	24.4	39.0	59.7	69.2	27.02
	% Cytotoxicity	83.6	69.2	58.6	43.8	35.6		89.7	75.6	58.4	36.5	29.4	

## Discussion

Over the last few years, significant number of researches have been dedicated to examining the impacts of naturally-occurring flavonoids in management and therapy of various types of cancer cells. Numerous studies have delved into this field, including the investigations conducted by Dias et al.^47^, Hazafa et al.^48^, Ravishankar et al.^49^, and Rupasinghe^50^.

The defatted extract, containing a high concentration of flavonoids from *S. japonicum* leaves was analyzed in the current study to quantify the total phenolics and flavonoids. The results revealed that the total phenolics content was 248.41 ± 0.23 mgGAE/g, while the flavonoids content was 735.21 ± 0.19 mg rutin/g. The high concentration of flavonoids in *S. japonicum* leaves necessitated the characterization of their constitutions. Through HPLC analysis, seven phenolic acids and six flavonoids were identified. Additionally, a comprehensive profile of flavonoids was revealed through negative ion mode UPLC/ESI–MS analysis. Thirty-three flavonoids were recognized, consisting of nine free aglycones, thirteen flavonoid mono glycosides, seven flavonoid diglycosides, two types of dihydroxyflavones, as well as an *O*-methylated isoflavone called biochanin A and its glucoside, sissotrin (biochanin A 7-*O*-*ꞵ*-D-glucoside). Furthermore, it was imperative to explore the fragmentations manner for these identified flavonoids aiming to obtain pertinent information regarding their structural characteristics.

Based on the literature review, it has been established that the primary fragmentation mechanism of the flavonoids aglycone is retro-Diels–Alder (RDA) reaction. This reaction is accompanied by the release of small neutral product ion and fragments such as CO_2_, CO, H_2_O, and C_3_O_2_^51^. Specifically, the most informative RDA fragments of flavonoids encompass the breaking of binary bonds in ring C. This results in the creation of 1,3A^−^ and 1,3B^−^ product ions, which afford valuable evidence about the substituents present in both A and B ring^52^. Those fragmentation peaks pay to unique identification for the flavonoids, with the 1,3A^−^ ion frequently being the predominant product ion detected in the negative acquisition mode.

The investigation of luteolin (compound 1, R_*t*_ 6.39) serves as an essential foundational study for our ongoing examination of flavonoid flavones. Luteolin, from a chemical perspective, is a phenolic molecule with hydroxyl groups, consisting of dual benzene rings (ring A and B) linked by γ-pyrone ring (ring C). The mass analysis of luteolin revealed the distinctive ions at *m/z*: 267, 257, and 241, which are produced through the loss of water, carbon monooxide, and two carbon dioxide, respectively. These ions are likely associated with the C ring^53^. Additionally, a smaller neutral loss corresponds to the cleavage of C_3_O_2_ (*m/z* 217), and this fragment ion further endures ketene loss, resulting in formation of the ion at *m/z* 175^54^.

**Figure 3:**
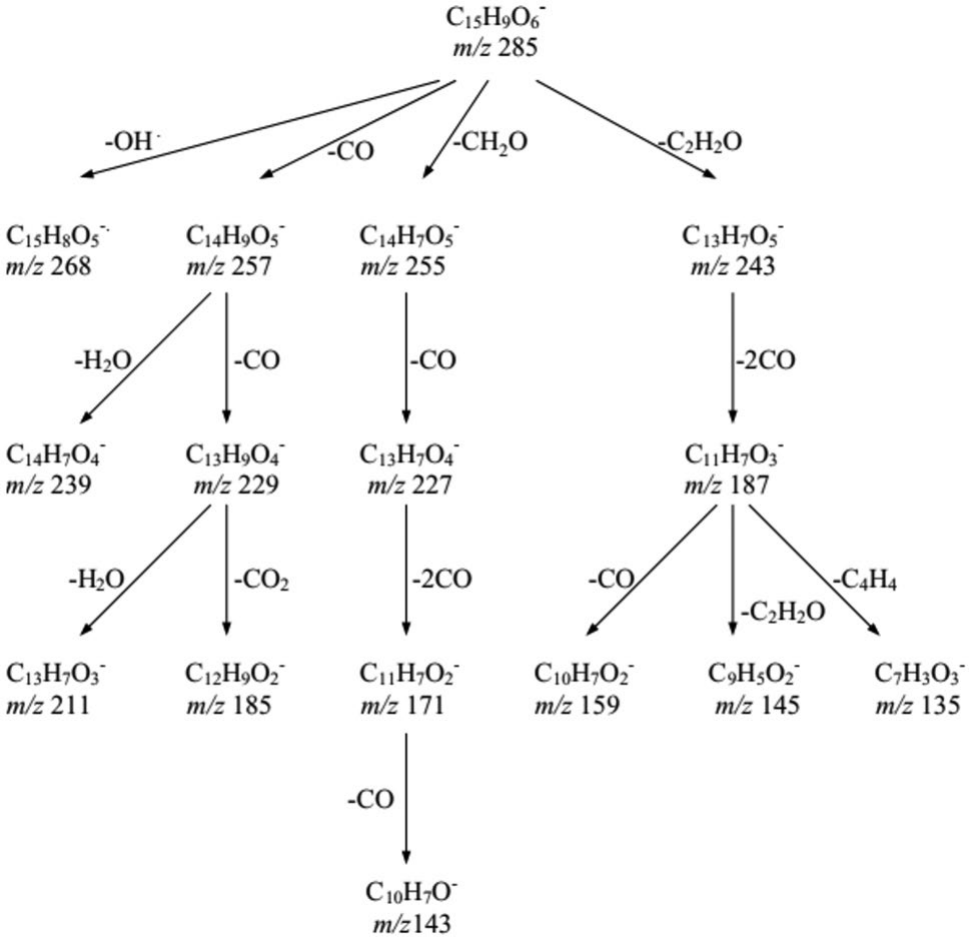
An illustrative diagram showcasing the suggested product ions generated through mass fragmentation for kaempherol.

Kaempferol, also known as 3,5,7,4'-tetrahydroxyflavone, compound 3, was selected as a representative example to illustrate the fragmentation series of flavonols. This particular compound, with a retention time of 9.21, exhibited [M − H]^−^ at *m/z* 285. The fragmentation process led to formation of four product ions, due to the removal of OH, CO, CH_2_O, and C_2_H_2_O groups, producing product ions with *m/z* values of 268, 257, 255, and 243, respectively^55^. Figure 3 presents a diagram showcasing the suggested product ions generated through mass fragmentation for kaempferol.

**Figure 4:**
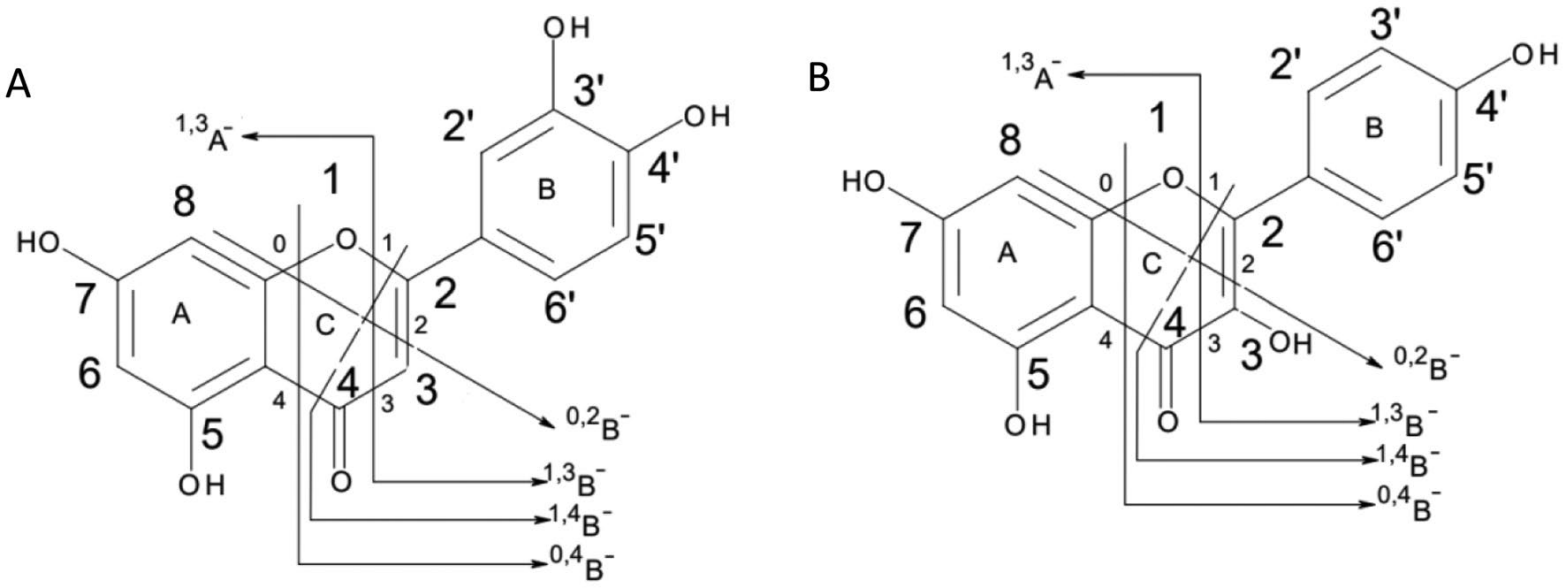
Proposed mass fragmentation patterns of luteolin flavone (**A**) and kaempherol flavonol (**B**).

In both of the aforementioned compounds, the fragment ions having *m/z* 151 &133 have been detected, which were suggested to be 1,3A^−^ and 1,3B^−^ ions, correspondingly^56^. However, the fragmentation pattern for 0,2A^−^ and 0,2B^−^ ions could distinguish between them due to the variation in the position of the hydroxyl group within their molecular structures. For luteolin, the hydroxyl group is situated in the B ring, the produced ions have been detected at *m/z* 148 & 136. On the other hand, in kaempherol, where the hydroxyl group is situated in the C ring, the corresponding product ions were *m/z* 164 & 120^55^. Figure 4 illustrates the distinct mass fragmentation patterns proposed for luteolin flavone and kaempherol flavonol.

For flavonoid *O*-glycosides, the sugar units and the free aglycone are obtained through a common fragmentation process. By analyzing the *m/z* values of consecutive product ions formed after *O*-glycosidic bonds breaking, the presence of hexoses and pentoses can be determined as [M-H-162] and [M-H-132], respectively. Additionally, further fragmentation of the aglycone [M-H]^−^ ion provides crucial evidence for accurate identification of flavonoids^57^.

**Figure 5:**
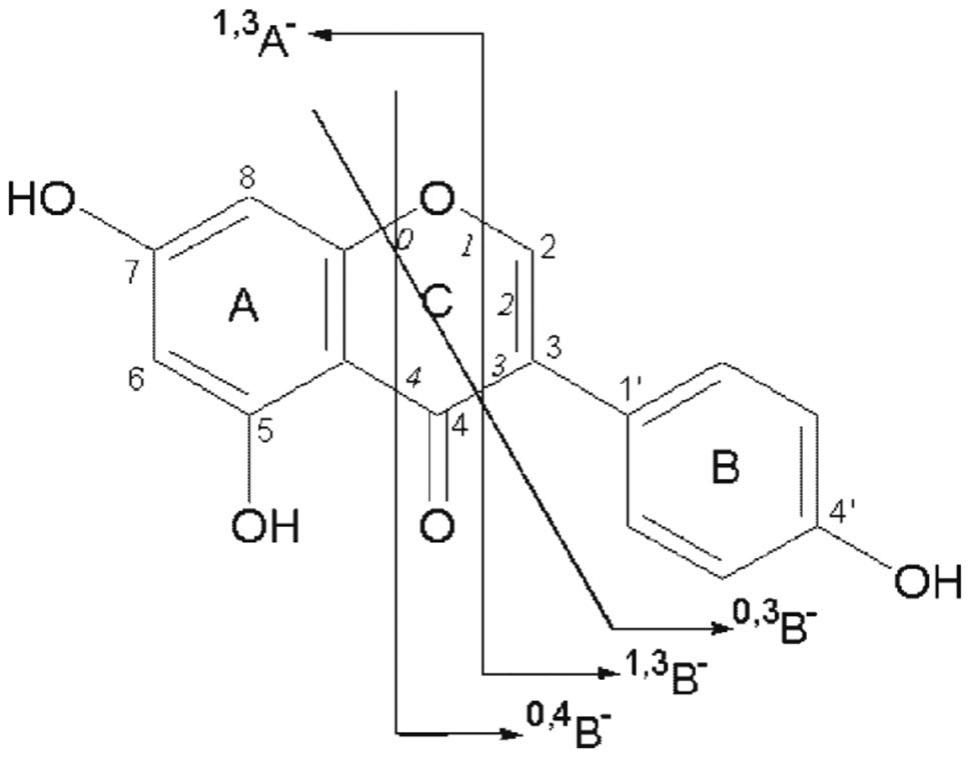
Proposed mass fragmentation patterns of genistein isoflavone.

For instance, compound 14, apigenin 7-*O-*glucoside, with a retention time of 15.05, and compound 25, genistein 7-*O*-glucoside, with R_*t*_ of 23.81, share the same [M-H]^-^ at *m/z* 431. Though, their differentiation becomes feasible by analyzing their fragmentation spectra^58,59^. Where apigenin generates a product ion at *m/z* 117, which corresponds to a 1,3B^−^ fragment, and another ion at *m/z* 151 for 1,3A^−^. Additionally, the loss of CO_2_ from the C ring is observed, resulting in an ion at *m/z* 225. Apigenin can further subsequently lose C_2_H_2_O and CO_2_, leading to the formation of ion at *m/z* 183. These fragmentation patterns align well with the previously reported data^53^. While the isoflavone genistein (compound 6, R_*t*_ 11.19) exhibits a structural difference from apigenin in terms of the positioning of the ring B. This disparity in structure can potentially result in distinct fragmentation behavior. The fragmentation paths of genistein lead to the release of carbon monoxide and carbon dioxide at ring C, attributed to [M–H–CO] (*m/z* 241) and [M–H–CO_2_] (*m/z* 225), separately. Additionally, a product ion with *m/z* 197 is observed, which is generated through the loss of CO_2_ from *m/z* 241, indorsed as [M–H–CO–CO_2_]. Subsequently, this product ion further undergoes fragmentation to yield another product ion with *m/z* 171, which is produced after C_2_H_2_ removal^59^. The proposed mass fragmentation patterns of genistein isoflavone are depicted in Fig. 5.

Moreover, the compound that was observed at a retention time of 15.35 has been identified as biochanin A, a methoxylated isoflavone. The mass fragmentation analysis revealed a product ion with *m/z* of 268 [M–H–CH_3_]. This finding is consistent with a previous study reported by Huck et al.^60^, which demonstrated that the loss of CH_3_ is a distinctive fragmentation pattern in methoxylated flavonoids. Additionally, the subsequent fragmentation of these radical anions readily resulted in the loss of a hydrogen atom, causing production of product ion with *m/z* 267. Furthermore, two other product ions with *m/z* 240 and 224 were also observed, corresponding to [M–H–CH_3_–CO] and [M–H–CH_3_–CO_2_], respectively. Notably, the two aforementioned product ions further underwent fragmentation, resulting in the generation of two product ions with *m/z* 239 and 223, respectively, through the loss of a hydrogen atom from each. It is worth mentioning that the mass fragmentations of other flavonoids can be found in various literature sources, including those by Guo et al.^61^, Justesen et al.^62^, and Horai et al.^63^. By surveying the cultivated varieties of *S. japonicum*, numerous flavonol glycosides were detected. The predominant flavonol glycosides identified were quercetin and kaempferol glycosylated at C-3 and C-7 with either glucose or rhamnose^16^. Furthermore, other various flavonol glycosides and abundant isoflavonoid glycosides mainly derived from the isoflavone genistein have been identified in the plant leaves, as genistein 7-*O*-*ꞵ*-D-glucopyranoside-4′-*O*-(6′′′-*O*-R-L-rhamnopyranosyl)-*ꞵ*-sophoroside and genistein 7-*O*-R-L-rhamnopyranoside-4′-*O*-(6′′′-*O*-R-L-rhamnopyranosyl)-*ꞵ*-sophoroside in addition to quercetin 3-*O*-*ꞵ*-D-glucopyranoside, and kaempferol 3-*O*-*ꞵ*-D-glucopyranoside^17^.

Among the nine species of *Styphnolobium* identified, only *S. japonicum, S. affine, S. burseroides,* and *S. monteviridis* were accessible for examination at the Kew Herbarium. The last two species exhibited glycosidic modifications of quercetin 3-*O*-rutinoside, kaempferol 3-*O*-rutinoside, and kaempferol 3-*O*-robinobioside that were akin to those found in *S. japonicum*^64^. On the other hand, *S. affine* demonstrated a different flavonoid profile characterized by a higher concentration of flavonol tri- and tetraglycosides. The major flavonoids identified in this species included quercetin triglycosides, kaempferol triglycoside, and kaempferol tetraglycoside^16^. The flavonol diglycosides detected in *S. affine* were characterized by the presence of glucose or rhamnose as the predominant sugar, rather than galactose, which was consistent with the findings in *S. japonica* (compounds 11,23,24,27,30,31,32). This suggests that the higher glycosides were derived from quercetin and kaempferol 3-*O*-glucosides. To sum up, the dissimilarity in leaf composition between *S. japonicum* and *S. affine* is of significant importance, as highlighted by Sousa and Rudd^64^. Nevertheless, it is worth noting that *S. bursero ides* and *S. monteviridis* exhibit comparable levels of leaf flavonoid chemistry to *S. japonicum*.

Two previously recognized compound; genistein 7-*O*-glucoside, and rutin were isolated, beside to kaempferol 3-*O*-*α*-L-rhamnopyranosyl-(1 → 6)-*β*-D-glucopyranosyl-(1 → 2)-*β*-D-glucopyranoside which isolated for the first time. The three isolated flavonoidal compounds were identified using a range of spectroscopic techniques, including UV, MS, ^1^H-NMR, and ^13^C-NMR.

The investigation of the biological aspect in the study revealed the defatted extract, containing a high concentration of flavonoids from *S. japonicum* leaves has exhibited remarkable abilities in terms of total antioxidant capacity and iron reduction. It has also displayed impressive effects in scavenging free radicals, surpassing the performance of ascorbic acid, which was employed as the benchmark. Furthermore, this extract has demonstrated noteworthy cytotoxic properties against a liver cell line (IC_50_ 337.9μg/ mL) and significant cytotoxicity against a lung cell line (IC_50_ 55.0 μg/ mL) as determined by the MTT assay. Moreover, the antioxidant capacities of the three isolated flavonoids have been evaluated, and it has been observed that their capability to scavenge the free radicals is concentration-dependent. Among the trio of flavonoids, rutin demonstrated the highest efficacy in scavenging DPPH and ABTS radicals, followed by genistein 7-*O*-glucoside, and finally kaempferol 3-*O*-*α*-L-rhamnopyranosyl-(1 → 6)-*β*-D-glucopyranosyl-(1 → 2)-*β*-D-glucopyranoside. Furthermore, the cytotoxicity percentages of the three isolated flavonoids on liver and lung cells were determined. In liver cells, genistein 7-*O*-glucoside, rutin, and kaempferol 3-*O*-*α*-L-rhamnopyranosyl-(1 → 6)- *β*-D-glucopyranosyl-(1 → 2)-*β*-D-glucopyranoside displayed cytotoxicity levels of 90.49%, 91.36%, and 82.25% at 100 ug/ml, respectively, resulting in IC50 values of 36.87%, 27.03%, and 36.89%, as compared to doxorubicin (IC50 27.65%). Conversely, in lung cells, the cytotoxicity of genistein 7-*O*-glucoside, rutin, and kaempferol 3-*O*-*α*-L-rhamnopyranosyl-(1 → 6)- *β*-D-glucopyranosyl-(1 → 2)-*β*-D-glucopyranoside was 86.03%, 83.45%, and 69.96% at 100 ug/ml, respectively, resulting in IC50 values of 30.63%, 25.51%, and 43.01%, in comparison to doxorubicin (IC50 27.02%).

It should be emphasized that the chemical structure of isolated flavonoids plays a crucial role in determining their biological properties^65,66^. The activity of these flavonoids varies depending on the substitution pattern, with the presence and position of hydroxyl groups, level of unsaturation, and number of substitutions significantly influencing their antioxidant capacity^67^. Numerous previous investigations have indicated that substitutions in ring A do not directly affect radical scavenging activity^68–70^. Instead, it is suggested that the nature of substitutions in ring B primarily determines the antioxidant efficacy of flavonoids. Additionally, the presence of a double bond between C2 and C3, along with the C=O group in ring C, enhances the free radical scavenging activity of unsaturated flavonoids when compared to saturated compounds^71^. Besides, Flavonoids have demonstrated their capacity to influence various pathways involved in the prevention and deceleration of cancer growth and metastasis. These pathways encompass acting as antioxidant to mitigate DNA injury, inhibition of growth factor receptors to diminish cell proliferation, facilitating DNA recovery, triggering apoptosis in cancer cells, and impeding tumor cell attack and angiogenesis through interfering with protein kinases and topoisomerases^72^.

*Genistin*, also known as genistein 7-*O*-*β*-D-glucoside, is believed to have numerous health impacts. It is recognized for its antioxidant, anticancer, and anti-inflammatory properties^73–76^. Various researches have demonstrated that genistin exhibits antioxidant activity by effectively scavenging DPPH and ABTS free radicals^77^. Additionally, Lai and Yen^78^ reported that genistin can efficiently scavenge peroxynitrite, which is significant in reducing the risk of cardiovascular conditions and inflammation-related diseases. Moreover, many in vitro preclinical investigations have shown that genistin holds promise in combating different types of human cancers^79,80^. Specifically, genistin has demonstrated its usefulness in the prevention and treatment of breast cancer cells by targeting signaling cascades, inducing apoptotic cell death, activating caspase-8/9, and cleaving poly (ADP-ribose) polymerase. A different in vitro investigation carried out by Phromnoi et al.^81^ demonstrated that genistin has the ability to hinder the activity of matrix metalloproteinase-3 and the invasion of cells in human invasive breast carcinoma, with the extent of inhibition being dependent on the concentration of genistin. Furthermore, genistin has exhibited promise in reducing the proliferation of human oral carcinoma, as indicated by Browning et al.^82^. In a separate in vitro study, Singh et al.^83^ provided evidence that treatment with genistin significantly impeded the growth of bladder cancer cells by arresting the cell cycle and inducing apoptosis.

*Rutin*, also known as quercetin 3-*O*-rutinoside, has been extensively studied for its pharmacological applications, primarily due to its antioxidant capacity. The antioxidant effects of rutin are achieved through various mechanisms, as demonstrated in several studies^84,85^. One of the mechanisms by which rutin exerts its antioxidant effects is through direct scavenging of reactive oxygen species, as highlighted by Enogieru et al.^86^. Rutin has the ability to neutralize ROS, thereby reducing oxidative stress and its associated damage. Another mechanism involves the modulation of cellular oxidative defense systems has been shown to increase the production of glutathione, an important antioxidant molecule, this was demonstrated by Kandemir et al.^87^. Furthermore, rutin exhibits xanthine oxidase inhibition, which is an enzyme involved in the generation of ROS. This mechanism was also elucidated by Enogieru et al.^86^. The aforementioned assumptions highlight rutin's potential as a promising compound for a wide range of therapeutic interventions.

Numerous researches have highlighted the significant role played by *Kaempherol glycosides* in acting as antioxidants and exerting antitumor effects on various cell lines, such as hepatic, colonic, and skin cell lines. The variations in antitumor activity could potentially be attributed to the remarkable ability of kaempherol to effectively inhibit AKT phosphorylation and induce the cleavage of caspase-9,-7,and-3, as demonstrated by Jung et al.^88^ and Wang et al.^89^. Moreover, Kluska et al.^90^ have provided evidence suggesting that kaempherol glycosides may indirectly affect the oxidative equilibrium by modulating the expression of antioxidant genes. According to a study conducted by Chen and Chen^91^, it was found that kaempherol glycosides have the ability to regulate various crucial components in cellular signal transduction pathways associated with apoptosis, angiogenesis, and metastasis. Additionally, similar to other flavonoids, kaempherol glycosides possess potent antioxidant properties and can effectively shield cells from oxidative stress, as reported by Kluska et al.^92^. Furthermore, the study accompanied by Imran et al.^93^ emphasized that kaempferol and its glycosides demonstrate antioxidant properties through various mechanisms, those encompass the elimination of harmful free radicals, hindrance of pro-oxidant enzymes, and stimulation of antioxidant enzymes. Consequently, this study accentuates the potential of flavonoids as a propitious pathway for the management of cancer.

## Conclusion

Although several studies have focused on the flavonoidal profile of *S. japonicum* leaves, kaempferol 3-*O*-*α*-L-rhamnopyranosyl-(1 → 6)-*β*-D-glucopyranosyl-(1 → 2)-*β*-D-glucopyranoside was isolated and identified for the first time from the plant leaves. The evaluation of the defatted extract with high flavonoid concentration against HepG2 and A549 cell lines is a novel approach, it is not particularly groundbreaking. Additionally, the antioxidant and anticancer properties of the isolated flavonoids were investigated for the first time in this study.

In details, the defatted extract, containing a high concentration of flavonoids from *S. japonicum* leaves demonstrated significant total antioxidant capacity, iron reducing power, and remarkable scavenging activities against DPPH and ABTS radicals. Additionally, it has exhibited noteworthy cytotoxic properties on liver (HepG2) and lung (A549) cell lines. The quantification and identification of phenolics and flavonoids in the defatted extract through HPLC and UPLC/ESI–MS analyses have conclusively demonstrated their substantial existence in the leaves of *S. japonicum* serving as crucial components for numerous biological processes. By conducting different spectral analysis, three flavonoids have been isolated and identified as genistein 7-*O*-*ꞵ*-glucoside, rutin, and kaempferol 3-*O*-*α*-L-rhamnopyranosyl-(1 → 6)-*β*-D-glucopyranosyl-(1 → 2)-*β*-D-glucopyranoside. The ability of these compounds to scavenge DPPH and ABTS free radicals, as well as cytotoxicity on liver and lung cells in concentration-dependent manner strongly indicates their potent activities. Therefore, this investigation underscores the potential of flavonoids as a beneficial avenue for the management of cancer. Future scientific studies should focus on investigating the specific protective and therapeutic properties of *S. japonicum* leaves as a valuable natural resource.

## Data Availability

All the data produced or examined throughout this study has been integrated into the content of this published article.

## Abbreviations

*S. japonicum*: *Styphnolobium japonicum*
HPLC: High-performance liquid chromatography
TLC: Thin layer chromatography
HepG2 cells: Human liver cancer cells
A549 cells: Adenocarcinomic human alveolar basal epithelial cells
IC_50_: Inhibitory Concentration 50
ND: Not detected
M.W.: Molecular Weight
M.F.: Molecular Formula
R_*t*_: Retention time
Rham: Rhamnose
Glu: Glucose
Genist: Genistein
Apig: Apigenin
Myr: Myricetin
Rut: Rutin
Soph: Sophoroside
RDA: Retro-Diels–Alder
MeOH: Methanol
NaOMe: Sodium methoxide
AlCl_3_: Aluminum chloride
HCl: Hydrochloric acid
NaOAc: Sodium acetate
H_3_BO_3_: Boric acid
ESI–MS: Electrospray ionization mass spectrometry
*m/z*: Mass-to-charge
ROS: Reactive oxygen species
